# Effect of Ge Content on the Formation of Ge Nanoclusters in Magnetron-Sputtered GeZrO_x_-Based Structures

**DOI:** 10.1186/s11671-017-1960-9

**Published:** 2017-03-16

**Authors:** L. Khomenkova, D. Lehninger, O. Kondratenko, S. Ponomaryov, O. Gudymenko, Z. Tsybrii, V. Yukhymchuk, V. Kladko, J. von Borany, J. Heitmann

**Affiliations:** 1 V. Lashkaryov Institute of Semiconductor Physics of NAS of Ukraine, 45 Pr.Nauky, 03028 Kyiv, Ukraine; 2 Institute of Applied Physics, TU Bergakademie Freiberg, D-09596 Freiberg, Germany; 3 Institute of Ion Beam Physics and Materials Research, Helmholtz-Zentrum Dresden-Rossendorf, D-01314 Dresden, Germany

**Keywords:** Germanium, Zirconium oxide, Nanoclusters, Phase separation, Magnetron sputtering, Thin films, X-ray diffraction, Ellipsometry, Raman scattering, Fourier Transform infrared spectroscopy, Auger electron spectroscopy

## Abstract

Ge-rich ZrO_2_ films, fabricated by confocal RF magnetron sputtering of pure Ge and ZrO_2_ targets in Ar plasma, were studied by multi-angle laser ellipsometry, Raman scattering, Auger electron spectroscopy, Fourier transform infrared spectroscopy, and X-ray diffraction for varied deposition conditions and annealing treatments. It was found that as-deposited films are homogeneous for all Ge contents, thermal treatment stimulated a phase separation and a formation of crystalline Ge and ZrO_2_. The “start point” of this process is in the range of 640–700 °C depending on the Ge content. The higher the Ge content, the lower is the temperature necessary for phase separation, nucleation of Ge nanoclusters, and crystallization. Along with this, the crystallization temperature of the tetragonal ZrO_2_ exceeds that of the Ge phase, which results in the formation of Ge crystallites in an amorphous ZrO_2_ matrix. The mechanism of phase separation is discussed in detail.

## Background

Germanium is compatible with current complementary metal oxide semiconductor (CMOS) technology. Physical scaling of bulk germanium to nanometer range reopened the route to novel applications. Germanium nanocrystals (Ge-ncs) can be used for electronic flash memories with improved write/erase speed as well as for optical devices and light emitters in visible and near-infrared spectral ranges.

Most of the research were performed on the Ge-ncs embedded in SiO_2_ [1–5], but a few studies of the Ge-ncs embedded in Al_2_O_3_ [6, 7] and HfO_2_ [8, 9] were done. Recently, the Ge-ncs embedded in ZrO_2_ [10, 11] and TaZrO_x_ [12] were investigated. However, for deeper understanding of the mechanism of the formation, growth, and crystallization of Ge-ncs in the ZrO_2_ matrix, further investigations are required.

It is well-known that monoclinic ZrO_2_ is the most stable crystalline phase at room temperature, while tetragonal and cubic crystal phases are stable at high temperatures [13]. From the microelectronic point of view, amorphous films as dielectrics are most attractive due to lower leakage current and better reliability properties in comparison to polycrystalline films. However, both tetragonal and cubic phases show much higher dielectric constants in comparison with amorphous one [14]. In this regard, it is important to stabilize tetragonal (cubic) ZrO_2_ films at room temperature.

To achieve the stabilization of these two phases at lower temperatures, their doping with aliovalent dopants (Y^3+^, Sc^3+^, Ca^2+^, Mg^2+^, Cu^2+^, etc.) is usually used. Such doping provokes the formation of oxygen vacancies for charge compensation that play an important role in stabilizing the cubic and tetragonal structures [15, 16]. However, the presence of additional oxygen vacancies will cause the formation of traps that affect the operation of the devices.

At the same time, doping with isovalent elements (Si, Ge, Ti, Sn, Ce, etc.) requires neither charge compensation nor oxygen vacancies’ formation. It was demonstrated experimentally that these dopants can stabilize tetragonal zirconia against monoclinic distortion but only some compositions were studied experimentally [17, 18]. Besides, it is known that ultrathin films (with the thickness of few nanometers) crystallize more often in tetragonal ZrO_2_ due to the stronger contribution of the surface energy to the free Gibbs energy or due to stress [19].

It was shown that the temperature of tetragonal-to-monoclinic transformation decreases with dopant concentration, while the crystallographic variations depend on dopant sizes. For instance, for large dopants as Ce^4+^, the *c*/*a* ratio of the tetragonal unit cell decreases with increasing Ce content, which causes the formation of cubic ZrO_2_ for higher Ce concentration. This behavior is similar to that observed in trivalent-cation-doped zirconia systems [16]. For small dopants as Ti^4+^ and Ge^4+^_,_ the *c*/*a* ratio increases with dopant content and these tetragonal solid solutions do not show any trend towards cubic phase formation. The stability of the tetragonal phase in Ge-doped ZrO_2_ films was explained by the formation of tetrahedral coordinated Ge with a Ge-O distance of 1.81 Å that is shorter than Zr-O bond (2.10 Å) [17]. Being stronger, Ge-O bond increases tetragonality of ZrO_2_ and stabilizes it.

It is worth to note that when ZrO_2_ was doped with group IV elements, solid solutions were considered to be constructed from oxide units as (MO_2_)_x_(ZrO_2_)_1-x_, where M = Ce, Ti, Ge [20, 21]. This did not assume the formation of Ge-ncs in Ge-doped ZrO_2_.

Recently, we have shown the formation of the Ge-ncs via phase separation of Ge-doped ZrO_2_ films [10]. However, the effect of Ge content on the Ge-ncs formation as well as the stability of the host oxide towards its thermal crystallization requires more consideration.

In this work, optical and structural properties of pure ZrO_2_ films and Ge-rich-ZrO_2_ layers with different Ge content produced by magnetron sputtering were studied with respect to deposition parameters and annealing treatment. The goal of this work was to find the ways to control the nucleation and crystallization of Ge-ncs independently from the crystallization of the high-k host material.

## Methods

### Sample Preparation

Radio frequency “top-down” magnetron co-sputtering setup equipped with 3 confocal sources (Ge, ZrO_2_ and SiO_2_) was used to grow the films on 6 inch substrates. These latter were p-type Si (100) wafers covered by 5-nm thermal SiO_2_. The sputtering was performed with pure Ar plasma (20 sccm flow) at room temperature on a rotating substrate (5 rotations per minute) allowed the deposition of homogeneous layer of the same thickness across the wafer. The power density (RFP) applied to the ZrO_2_ target was fixed at RFP_ZrO2_ = 3.3 W/cm^2^. To achieve different Ge content in the films, the power density applied to the Ge target was varied from RFP_Ge_ = 0.7 to 2.2 W/cm^2^. For comparison, pure Ge and pure ZrO_2_ films were deposited in addition. The film-thickness was fixed at about 500 nm, which was achieved by adjusting the deposition time. More details can be seen elsewhere [10, 11].

**Fig. 1:**
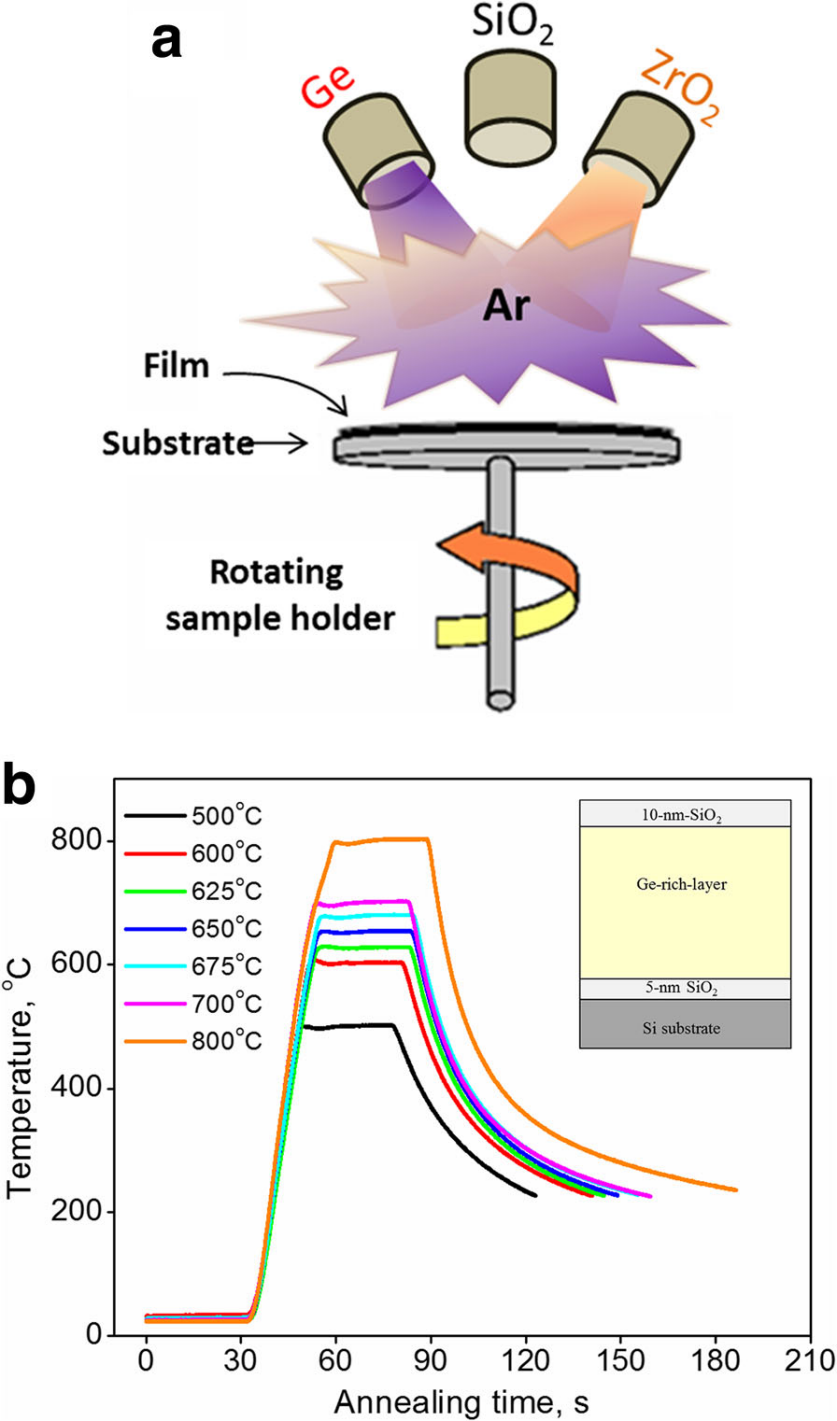
Schematic presentation of “top-down” co-sputtering process (*left image*), sample architecture and annealing parameters (*right image*)

All films were covered with a 10 nm SiO_2_ capping layer, which was sputtered from a SiO_2_ target with RFP_SiO2_ = 3.3 W/cm^2^. This manipulation was used to prevent Ge outward diffusion in Ge-doped samples upon thermal treatment [22]. After deposition, each 6-in wafer was cut into small pieces of 1 × 1 cm^2^ (so-called hereafter as “samples”). Both schematic presentation of deposition process and the architecture of the samples are shown in Fig. 1.

To study the effect of thermal treatment on the sample properties, the samples were subsequently annealed at T_A_ = 500–800 °C for 30 s in nitrogen flow using a rapid thermal processing tool. The temperature profiles are presented in Fig. 1.

### Sample Characterization

As-deposited and annealed samples were characterized with Fourier-transform infrared (FTIR) spectroscopy, multi-angle laser ellipsometry, Auger electron spectroscopy, Raman scattering and X-ray diffraction.

FTIR spectra were measured in the range of 460–4000 cm^−1^ by means of a Spectrum BX FTIR spectrometer (PerkinElmer Inc.) and a Nicolet Nexus FTIR spectrometer. The spectra were recorded in “transmission” mode at normal or Brewster (65°) incidence of excited light, using both atmospheric and Si substrate corrections. Multi-angle laser ellipsometric measurements were carried out with a LEF-3 M setup operating with a 632.8-nm light wavelength for the range of incidence angles of 45–90°. More details can be seen elsewhere [23].

X-ray diffraction data were collected with a Philips X'PERT apparatus using Cu K_α_ radiation in a 2θ range of 20°–80°. An asymmetric grazing geometry was chosen to increase the volume of material interacting with the X-ray beam, as well as to reduce contributions from the Si substrate. The data were compared with standard cards of Powder Diffraction File Database (#37-1484 for monoclinic ZrO_2_, #50-1089 for tetragonal ZrO_2_, and #4-0545 for cubic Ge).

Raman spectra were excited with 488.0 nm radiation of an Ar^+^-laser and recorded using a LabRam HR800 micro-Raman system in backscattering mode. The power of the laser excitation was chosen to prevent the heating of the samples.

The stoichiometry of the films was determined by Rutherford backscattering spectrometry (RBS) and Auger electron spectroscopy (AES). For RBS study, the films were deposited on carbon substrates at the same deposition conditions as described above. The RBS measurements were carried out using He^+^ ions with energy of 1.7 MeV and a backscattering angle of 170°. For AES experiment, the Auger microprobe JAMP 9500 F (JEOL), with 3 nm resolution in the secondary electron image mode was used. The microprobe was equipped with sensitive hemispheric Auger spectrometer with energy resolution ΔE/E from 0.05 to 0.6% and an ion etching gun for layer-by-layer analysis with diameter of Ar^+^ ion beam 120 μm, able to move by raster 1 × 1 mm. Variation range of the beam Ar^+^ ion energy is from 0.01 to 4 keV, while minimal beam current is 2 μA with 3 keV. More details can be found elsewhere [24].

All the measurements were performed at room temperature.

## Results and Discussion

### Ellipsometry, RBS, and AES Study

One of the most effective optical methods of researching the properties of the interface of two media and thin film heterostructures is ellipsometry. Both the thickness and optical constants of layers can be determined, when two quantitative characteristics (amplitude ratio Ψ and phase difference Δ) of polarized light reflected from the surface are examined simultaneously. According to the ellipsometric measurements of the polarization angles Ψ(φ) and Δ(φ), the refractive index *n*, absorption coefficient *α* and thickness *d* of the film can be extracted by solving the inverse ellipsometric task using the method of minimizing the quadratic objective function [25].

**Fig. 2:**
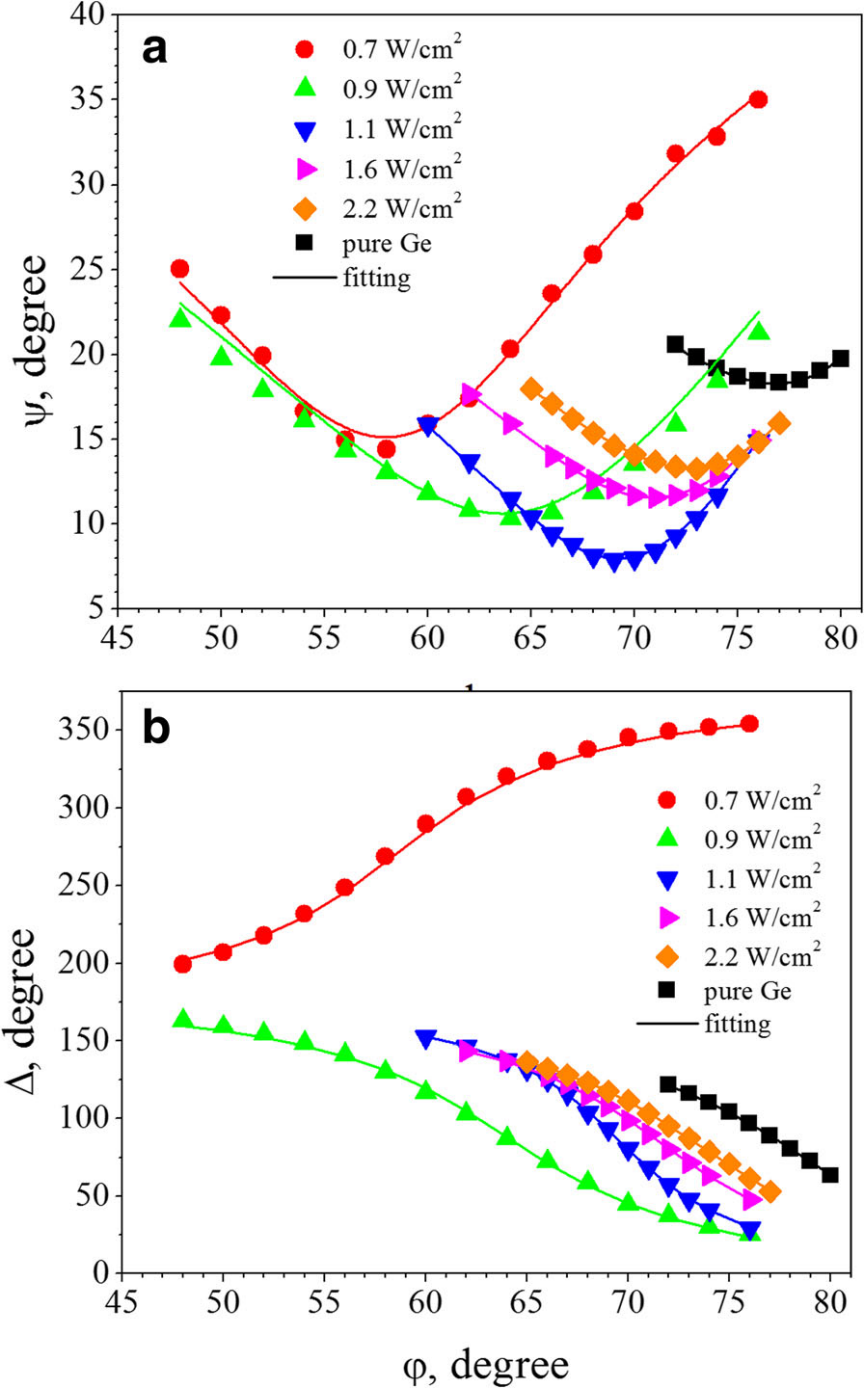
Experimental data (*symbols*) and fitting curves for Ψ(φ) (**a**) and Δ(φ) (**b**) obtained for the Ge-ZrO_2_ samples grown with different RFP_Ge_. The RFP_Ge_ values are shown in the *graphs*

**Fig. 3:**
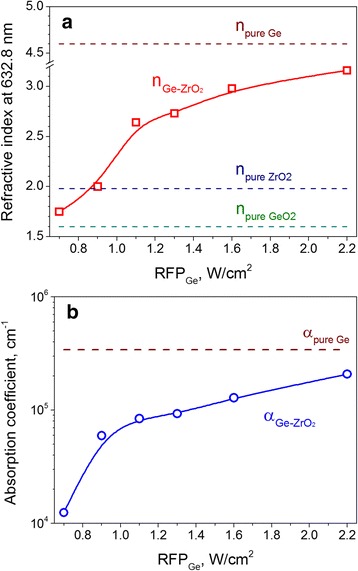
Variation of the refractive index (**a**) and absorption coefficient (**b**) for the Ge-ZrO_2_ samples versus RFP_Ge_. The *dashed lines* showed the values corresponded to pure Ge and pure ZrO_2_ samples as well as tabulated data for GeO_2_ for comparison

The experimental data of Ψ(φ) and Δ(φ) obtained for the Ge-ZrO_2_ samples deposited with different RFP_Ge_ are shown in Fig. 2(a,b). To fit these data, a four-phase optical model was applied [25, 26]. It consists of a silicon substrate, thermal SiO_2_ layer (with the thickness of about 5 nm appeared on Si substrate surface due to wafer processing); non-stoichiometric Ge-ZrO_2_ layer; capping SiO_2_ layer and a surface rough layer that is composed of a mixture of void space and SiO_2_ capping layer. Figure 3 shows the variation of *n*_Ge-ZrO2_ and *α*_Ge-ZrO2_ for as-deposited Ge-ZrO_2_ samples together with the data obtained for pure Ge and pure ZrO_2_ films.

Figure 3 shows the evolution of the refractive index *n*_Ge-ZrO2_ and the absorption coefficient *α*_Ge-ZrO2_ for Ge-ZrO_2_ samples sputtered at various RFP_Ge_. Generally, the increase of both parameters with RFP_Ge_ can be seen. However, two specific ranges of the *n*_Ge-ZrO2_ variation can be distinguished when this latter is compared with the refractive index of ZrO_2_, i.e., *n*_Ge-ZrO2_ < *n*_ZrO2_ for the films grown with RFP_Ge_ < 0.9 W/cm^2^ and *n*_Ge-ZrO2_ > *n*_ZrO2_ when RFP_Ge_ > 0.9 W/cm^2^. For the latter case, the *n*_Ge-ZrO2_ increases from 2.64 (RFP_Ge_ = 1.1 W/cm^2^) to 3.16 (RFP_Ge_ = 2.2 W/cm^2^). Since the refractive index of pure Ge (*n*_Ge_ = 4.60) exceeds that value of pure ZrO_2_ (*n*_ZrO2_ = 1.98), this tendency is in agreement with the higher Ge content in the films grown with higher RFP_Ge_.

Taking into account Bruggeman effective medium approximation and considering the Ge-ZrO_2_ samples as a mixture of pure Ge and pure ZrO_2_ phases, i.e., Ge_x_(ZrO_2_)_1-x_, the Ge content can be roughly estimated. More details about this procedure can be found in [10]. The obtained results show that the Ge content could be adjusted over a wide range (up to 47 at%) via the RFP_Ge_ variation, while keeping other deposition parameters constant (Table 1). It is worth to note that any contribution of GeO_2_ or voids was ruled out. This can result in the underestimation of Ge content in the films grown with RFP_Ge_ = 1.1–2.2 W/cm^2^.

**Table 1:** The parameters of the samples versus deposition conditions

RFP_Ge_, W/cm^2^	Ellipsometry data		RBS data^a^				
	n @ 632.8 nm	Ge, at.%	Ge, at%	Zr, at %	O, at%	Si, at%	Density, at/cm^2^
0	1.970	0	0	26.5	67.0	6.0	1.35E + 18
0.7	1.749	~17	15.0	17.5	62.9	4.0	1.43E + 18
0.9	2.000	~21	(22.0)	(16.0)	(57.0)	(3.0)	-
1.1	2.641	~30	30.0	14.0	52.0	3.0	1.20E + 18
1.3	2.730	~32	(33.0)	(13.0)	(50.0)	(3.0)	-
1.6	2.983	~40	42.0	10.5	43.9	2.5	1.17E + 18
2.2	3.167	~47	53.0	8.0	35.9	2.0	1.09E + 18

^a^Note. The content of the elements placed in the parentheses was obtained by the extrapolation of RBS data

The samples grown with the RFP_Ge_ = 0.7 W/cm^2^ show a refractive index of *n*_Ge-ZrO2_ = 1.75. Such a low value supposes the formation of a phase with lower refractive index, for instance, GeO_2_ or Ge suboxides. Assuming the Ge-rich-ZrO_2_ film is a mixture of GeO_2_ and ZrO_2_ phases, the Ge content of this sample was estimated to be about 17%.

The determination of the Ge content for the sample grown with RFP_Ge_ = 0.9 W/cm^2^ was more complicated. As one can see from Fig. 3a, this sample has a refractive index of *n*_Ge-ZrO2_ = 2.0 which is close to *n*_ZrO2_ = 1.98. Taking into account the results described above, as well as the higher absorption coefficient measured for this sample (Fig. 3-b), one can assume that it should contain a Ge content higher than 17% obtained for the sample fabricated with RFP_Ge_ = 0.7 W/cm^2^. However, the consideration of this sample either as Ge_x_(ZrO_2_)_1-x_ or as (GeO_2_)_x_(ZrO_2_)_1-x_ did not bring any reasonable values for the Ge content. Nevertheless, taking into account the variation of Ge content obtained for all other samples, one can extrapolate the Ge content for the sample grown with RFP_Ge_ = 0.9 W/cm^2^. Under these assumptions, it turned out that this sample contains about 21 at % of germanium.

Some of the samples described above were characterized by RBS and AES. The results on RBS are summarized also in Table 1. These data are in good agreement with those extracted from ellipsometry. However, some Si contamination at the level of 2–4 at% was detected. It could appear due to cross contamination of Si deposition processes carried out earlier. However, Si content decreases with increasing RFP_Ge_ (Table 1).

**Fig. 4:**
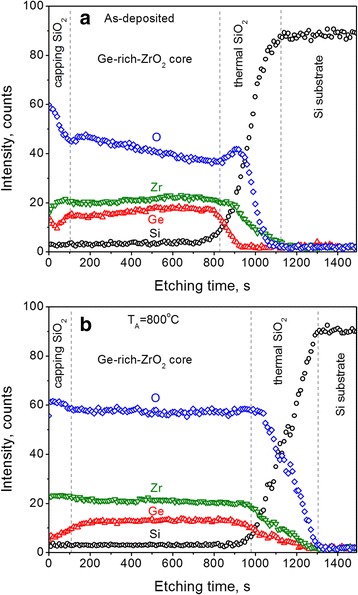
Element depth profiles in the Ge-ZrO_2_ sample grown with RFP_Ge_ = 1.1 W/cm^2^ obtained for as-deposited (**a**) and annealed at 800 °C (**b**) samples

The analysis of the samples with AES showed a homogeneous distribution of Ge and Zr in the Ge-rich-ZrO_2_ volume (Fig. 4a). At the same time, some incorporation of Zr and Ge in the thermal and capping SiO_2_ layers was observed (Fig. 4a) that can be due to preferential sputtering at the interfaces. Besides, similar to RBS experiment, some traces of Si were also detected. It is worth to point that doping with Si is considered as a way to stabilize amorphous structure of HfO_2_ and ZrO_2_ films [26, 27]. However, in this case, Si content should be higher than 10%. This means that Si contamination observed in our ZrO_2_ and Ge-rich ZrO_2_ samples is negligible to affect significantly their structural properties.

After thermal treatment the homogeneous distribution of Ge and Zr was conserved (Fig. 4b). However, some decrease of Ge content in the volume of Ge-rich-ZrO_2_ was observed, whereas the near surface region was found to be depleted in Ge. This transformation of Ge distribution can be caused by the outward diffusion of Ge during annealing (Fig. 4b).

To investigate the effect of Ge content on the microstructure of the Ge-rich-ZrO_2_ films and on its evolution with annealing, the samples described above as well as pure ZrO_2_ films were investigated by FTIR.

### FTIR Study of Pure and Ge-Rich ZrO_2_ Materials

Among express and nondestructive methods, FTIR holds an important place. It allows the evolution of the samples’ microstructure to be monitored as a function of the chemical composition and/or thermal treatment. In regard to Ge-rich-ZrO_2_ materials, only a few groups reported about FTIR studies of bulk ZrGeO_4_ [28] or GeO_2_ contained glasses [29]. At the same time, the Ge-rich-ZrO_2_ thin films were not often addressed [10, 18]. Nevertheless, the interpretation of experimental FTIR spectra can be based on the comparison of infrared spectra of ZrO_2_ [30] and GeO_2_ and their transformation with the increase of the contribution of high-k phase in solid solution. For our samples this means that we will compare FTIR spectra measured for pure ZrO_2_ films with those obtained for Ge-rich-ZrO_2_ counterparts, as well as with data available in the literature (Table 2). The validity of such an approach was demonstrated for Si-rich HfO_2_ [26, 31], Ge-rich HfO_2_ thin films [8], and Zr-doped Ta_2_O_5_ [32].

**Table 2:** Assignment of Zr-O and Si-O related vibration bands

Type of bonding	Spectral position, cm^−1^ (vibration type)	Reference
Zr-O monoclinic	350,425,520,595,740 (as-deposited) (20 °C) 335,410,505,575,740 (shoulder) (673 °C) 325,400,505,575 (910 °C)	[30]
Zr-O tetragonal	485, 615	
Zr-O cubic	450, 485, 615	
Si-O-Si	470 (rocking) (TO_1_), 820 (bending) (TO_2_) 1086 (asymmetric) (TO_3_)	[33]
Si-O^1−^	970 (terminal Si-O groups produced by network disruption)	[34]

#### Pure ZrO_2_

Usually, transmission FTIR spectra are detected under normal incidence of exciting light. However, when Brewster configuration is used, additional longitude phonons can be revealed. For pure ZrO_2_ films, transmission FTIR spectra show the vibration band in the range of 380–800 cm^−1^. For amorphous films, this band is broad and featureless. For ZrO_2_ crystalline films, several bands can be detected (Table 2). The appearance of a vibration band peaked at 740–770 cm^−1^ is the evidence for the formation of monoclinic ZrO_2_.

**Fig. 5:**
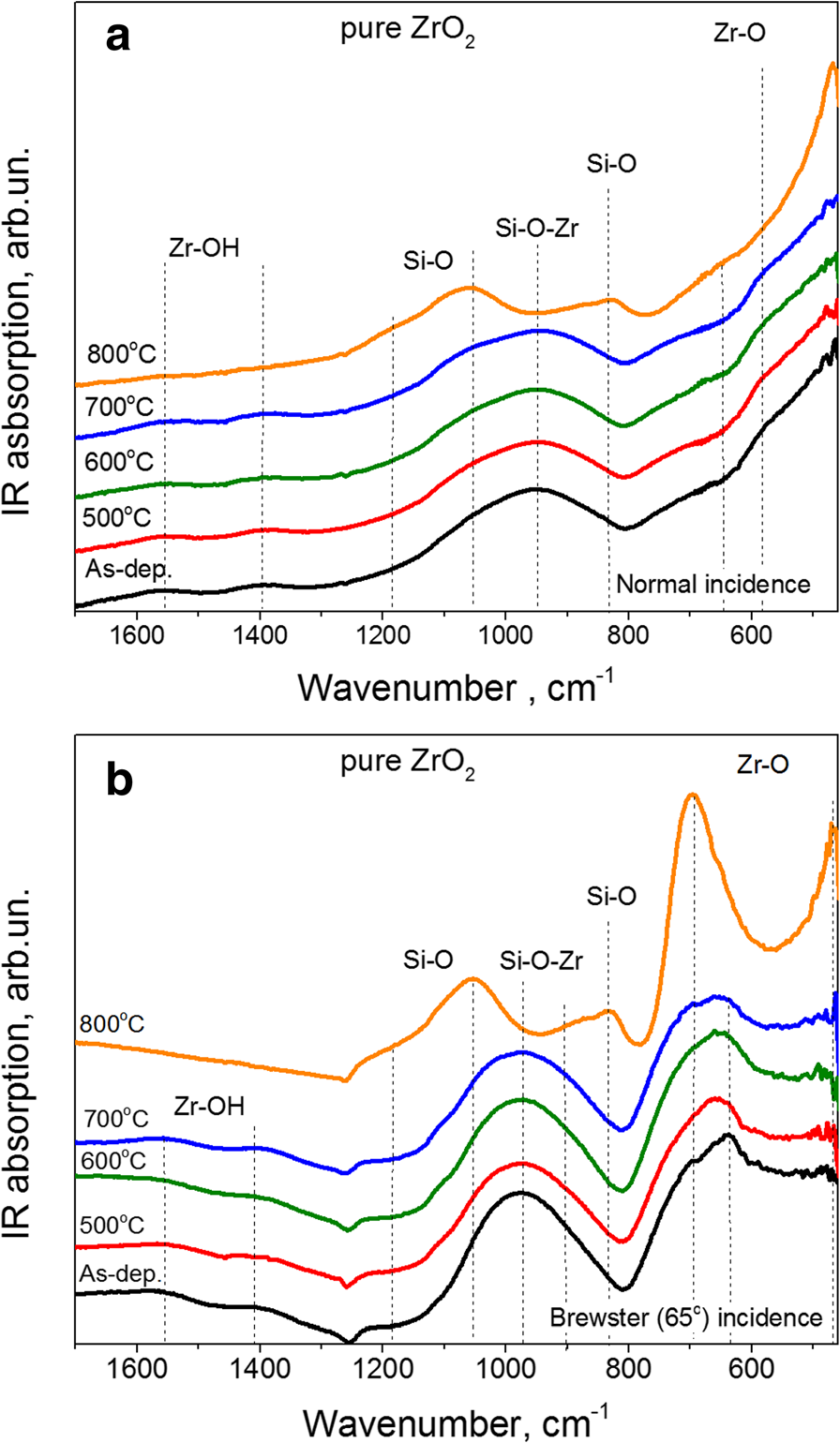
Evolution of FTIR spectra with annealing temperature for ZrO_2_ films measured under normal (**a**) and Brewster (**b**) incidence of excited beam light. Annealing temperatures are mentioned in the figures

FTIR spectra of as-deposited and annealed pure ZrO_2_ films are shown in Fig. 5a and b, respectively. Two broad bands in the range of 400–750 cm^−1^ and 800–1250 cm^−1^ can be seen. Since both bands are featureless, this means that as-deposited ZrO_2_ films are amorphous.

Annealing of pure ZrO_2_ samples at T_A_ ≤ 700 °C did not cause the transformation of FTIR spectra. When T_A_ = 800 °C, the Zr-O related band becomes narrower resulting in the appearance of the bands peaked at ~460 cm^−1^ and at ~700 cm^−1^ as well as a shoulder at about 610–620 cm^−1^ (Fig. 5a,b) corresponding to Zr-O vibrations. Since the peak related to monoclinic ZrO_2_ was not detected for the annealed films, one can suppose an appearance of tetragonal or cubic ZrO_2_ after annealing (Table 2).

Another vibration band was detected in the range of 1000–1200 cm^−1^ (Fig. 5a,b). It peaks at about 960–980 cm^−1^. Taking into account the architecture of the samples (Fig. 1), the presence of Zr ions inside SiO_2_ interfacial and capping layers as well as the presence of contaminated Si ions in ZrO_2_ core (Fig. 4), we can attribute the band peaked at 960–980 cm^−1^ to Si-O-Zr vibrations. This band is still stable upon annealing at T_A_ ≤ 700 °C. For T_A_ = 800 °C, the transformation of Si-O-Zr band occurs via the appearance of the bands peaked at ~820 cm^−1^, ~1060 cm^−1^, and ~1160 cm^−1^ (shoulder) related to Si-O vibrations [33, 34] (Table 2). At the same time, some contribution of the Si-O-Zr band is still visible for the samples annealed at T_A_ = 800 °C (Fig. 5a,b). Besides, two weaker bands peaked at about 1400 cm^−1^ and 1600 cm^−1^ were detected for as-deposited ZrO_2_ films and those annealed at T_A_ ≤ 700 °C. Both of these bands are related to Zr-OH vibrations due to presence of moisture in the film, which contribution decreases with rising T_A_.

#### Ge-Doped ZrO_2_ Samples

Usually, a triplet in the range of 515, 555, and 587 cm^−1^ connected with hexagonal GeO_2_ phase can be observed for Ge-O bonds. In the case of glassy Ge oxide, this triplet reconstructs in one broad band [35, 36]. For ZrGeO_4_, the position of Ge-O vibration bands can be detected at 453, 553 and 696 cm^−1^ (Table 3). Besides, the band at 860, 910, 1060 and 1080 cm^−1^ can be also originated by Ge-O bonds in crystalline material [28, 29, 37].

**Table 3:** Assignment of Ge-O related vibration bands

Type of bonding	Spectral position, cm^−1^ (vibration type)	Reference
Ge-O	515, 555 and 587 (triplet of hexagonal GeO_2_) (stretching)	[35, 36]
Ge-O-Ge	580 (bending), 870 (stretching)	[37]
Ge-O^−^	1060-1080 (non-bridging)	[37]
Ge-O	696 stretching (in ZrGeO_4_)	[28]
Ge-O-Ge	575 bending (in ZrGeO_4_)	
O-Ge-O	453 bending (in ZrGeO_4_)	
Ge-O	410 (ν(M-O) in [MO_6_])	[29]
	453 (δ(Ge-O) in [GeO_4_])	
	506 (ν(Ge-O) in [GeO_4_] glassy GeO_2_)	
	502-580 shoulder (ν(Ge-O) in glassy GeO_2_)	
	575 (ν(Ge-O) in [GeO_6_])	
	586 (δ(Ge-O) in [GeO_4_])	
	696 (ν(Ge-O) in [GeO_4_]^3−^ in orthogermanates)	
	doublet 773, 793 (ν(Ge-O) in metagermanates [GeO_3_]^2−^)	
	shoulder 790–890 (ν(Ge-O) in polygermanates)	
	910 (ν(Ge-O) in [GeO_4_])	
	1060 (ν(Ge-O) in orthogermanates [GeO_4_]^2−^)	
	1080 (ν(Ge-O) in orthogermanates [GeO_4_]^3−^)	

In the case of Ge-rich-ZrO_2_ films, besides Zr-O and Ge-O bond, one can expect the incorporation of Ge into Zr-O-Zr bond and an appearance of the Zr-O-Ge band. The peak position of the latter one should be observed at higher wavenumbers than that of Zr-O-Zr band. This assumption is based on the fact that the vibration frequency is reciprocal to the masses of bonding ions and m_Ge_ < m_Zr_.

**Fig. 6:**
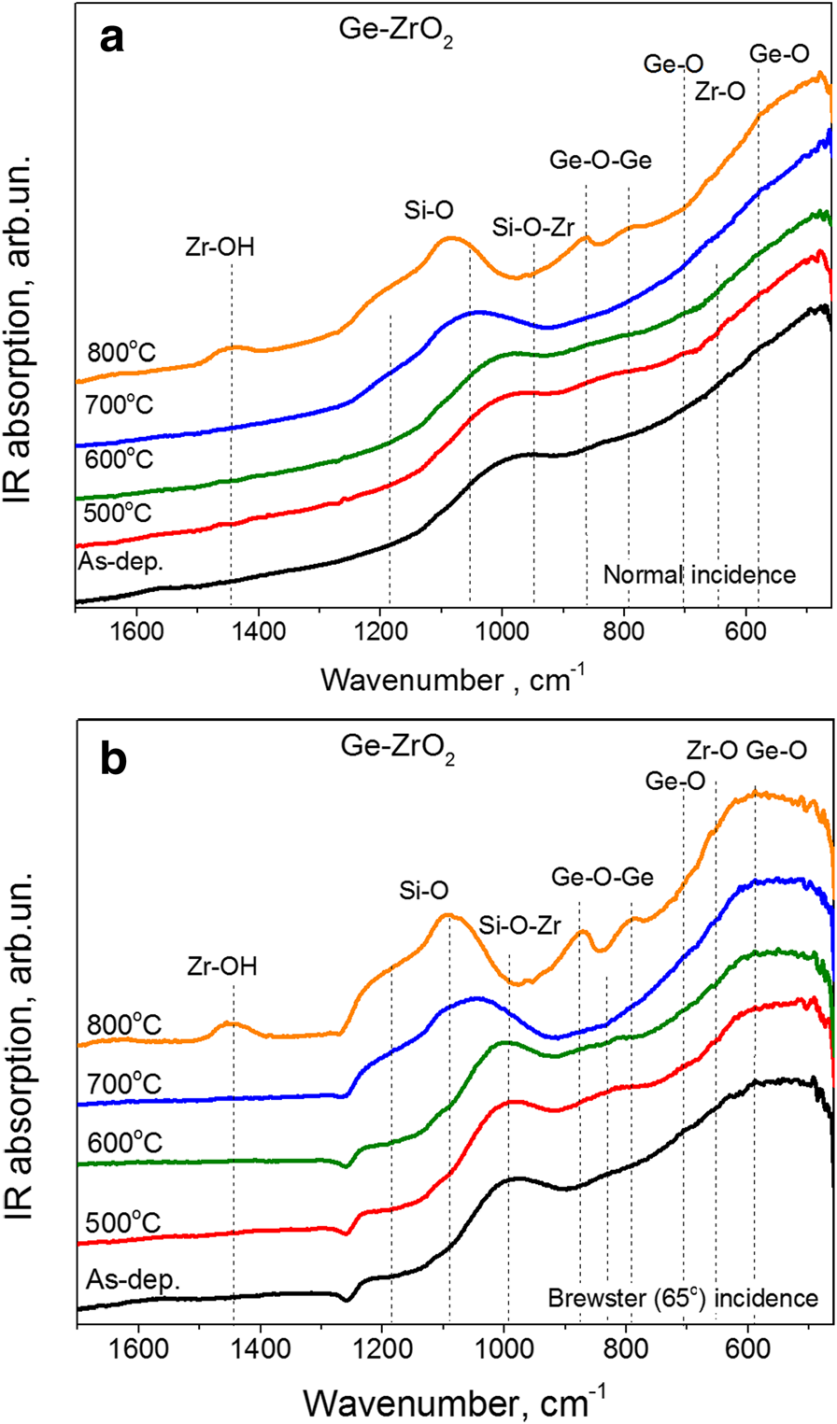
Evolution of FTIR spectra with annealing temperature Ge-rich-ZrO_2_ films grown with RFP_Ge_ = 0.9 W/cm^2^ and measured under normal (**a**) and Brewster (**b**) incidence of exciting beam light. Annealing temperatures are mentioned in the figures

Figure 6 shows the FTIR spectra of as-deposited Ge-rich-ZrO_2_ samples grown with RFP = 0.9 W/cm^2^ as well as their evolution with annealing treatment. The spectra of as-deposited sample contain two broad bands in the range of 450–800 and 900–1000 cm^−1^. The featureless shape of these bands testifies to the amorphous nature of the films. It is worth to note that the higher Ge content in the as-deposited Ge-rich ZrO_2_ films, the more featureless and broader FTIR spectra were observed.

The band peaked at about 990–1000 cm^−1^ can be attributed to the Si-O-Zr vibrations similarly to the case of ZrO_2_ samples described above. However, this band can be a superposition of Si-O-Zr and Si-O-Ge vibrations because the presence of both Ge and Zr ions was seen in SiO_2_ interfacial and capping layers as well as some Si contamination was detected for Ge-rich-ZrO_2_ core of the sample.

Another broad band appeared in the range of 450–800 cm^−1^ (Fig. 6). The comparison of this band with that of pure ZrO_2_ films allows its broadening to be ascribed to the Ge incorporation in ZrO_2_ host (Fig. 5).

The FTIR spectra of the Ge-ZrO_2_ films annealed at T_A_ = 500 and 600 °C are similar to the ones of as-deposited films. This proves the stability of the microstructure of the samples. Annealing at T_A_ = 700 °C leads to the transformation of Si-O-Zr (Si-O-Ge) bands and a shift of its peak position to ~1045 cm^−1^ as well as an appearance of the shoulder at ~1100 and 1180 cm^−1^ that is mainly due to Si-O vibrations (Fig. 6). For T_A_ = 800 °C, this transformation is significant. The Ge-O bands peaked at about 790 and 870 cm^−1^ are clearly seen. Besides the increase of the intensity at about 600 cm^−1^ can be due to the overlapping of Zr-O (615 cm^−1^) and Ge-O (595 cm^−1^) vibration bands.

It is worth to note that the presence of OH-related band peaked at ~1440 cm^−1^ was detected for the films annealed at T_A_ = 800 °C. This can be explained by the adsorption of water by the surface of annealed films. These OH groups can be linked with Zr ions as shown in Fig. 6, but the appearance of Ge-OH band cannot be ruled out.

Thus, the evolution of FTIR spectra described above confirms the phase separation process in Ge-rich-ZrO_2_ films and the formation of Ge clusters can be expected. Since such formation can be revealed rather by Raman scattering and XRD methods than FTIR ones, same samples were investigated by additional techniques.

### Raman Scattering Spectra of Ge-Rich ZrO_2_ Materials

The spectral positions and full-widths of Ge-related phonon modes depend on the material structure. The transition from the amorphous to the crystalline state leads to a significant narrowing of the phonons and to a shift of peak positions towards higher wavenumbers.

Usually, amorphous Ge materials show peaks at about 275 cm^−1^ (TO), 200 cm^−1^ (LO-LA), and 80 cm^−1^ (TA) [38]. Recently, it has been shown that these bands can be distinguished not only for pure Ge films, but also for Ge-doped high-k oxides [8–10]. Thus, one can expect to observe several peaks in the range of 50–400 cm^−1^ in our Ge-rich samples.

**Fig. 7:**
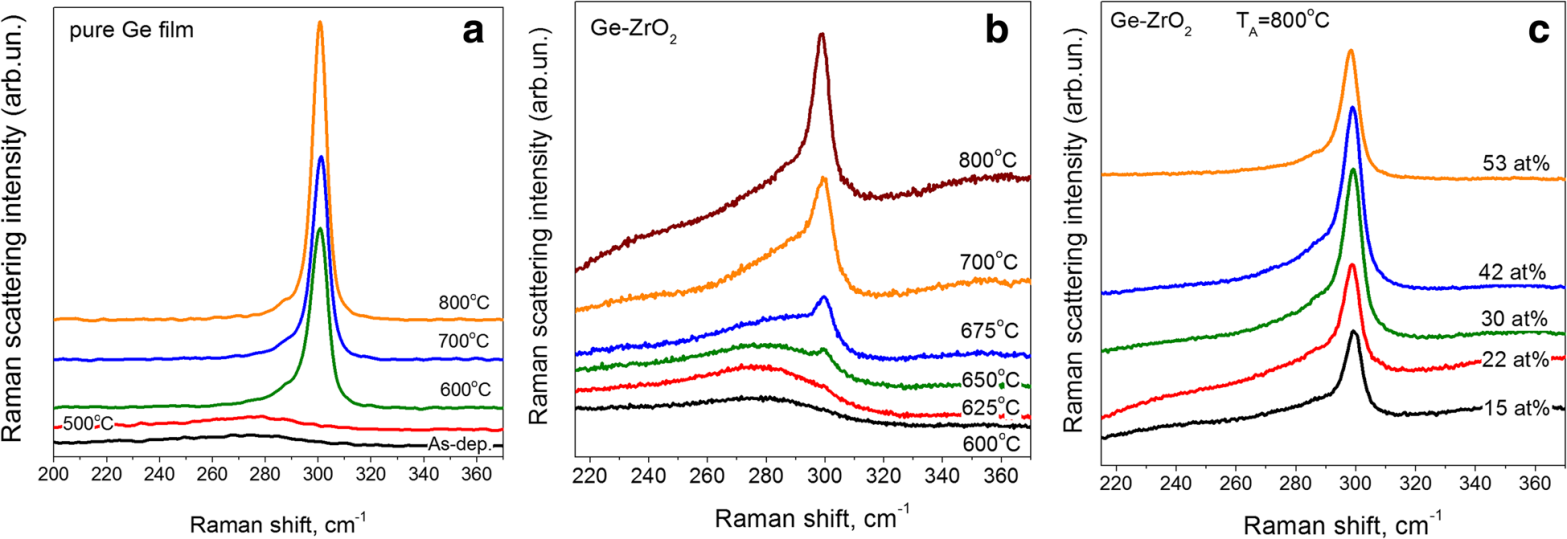
Evolution of Raman scattering of pure Ge (**a**) and Ge-rich-ZrO_2_ (**b**, **c**) samples with annealing temperature (**a**, **b**) and Ge content in the films (**c**). For (**b**) RFP_Ge_ = 0.9 W/cm^2^, [Ge] ~ 22%

Raman scattering spectra of as-deposited pure Ge films were found to be broad and featureless with the peak position at about 275–277 cm^−1^ that testifies to amorphous structure of the films (Fig. 7a). Annealing of the films at T_A_ = 500 °C did not change the film structure, while for higher T_A_ the presence of a sharp Ge peak at about 299 cm^−1^ was detected due to the crystalline phase. Its intensity increases with rising T_A_ accompanied by a band narrowing (Fig. 7a). Based on these data one can conclude that the crystallization of sputtered Ge films sets in at T_A_ = 550–600 °C.

Raman scattering of as-deposited Ge-rich-ZrO_2_ samples were found to be broad and featureless with a maximum at 272–275 cm^−1^ (Fig. 7b). Whatever Ge content in the films, thermal treatment at T_A_ ≤ 600 °C causes negligible transformation of the spectra shape. Annealing of Ge-rich-ZrO_2_ films results in the narrowing of the spectrum as well as in the appearance of a small peak at ~298 cm^−1^_,_ due to the crystalline phase (Fig. 7b). The intensity of this band increases significantly with T_A_ rise up to 800 °C, giving an evidence of Ge phase crystallization.

It is worth to note that for the higher Ge content, the formation of Ge clusters and their crystallization occurs at lower T_A_. For the same T_A_ values, the Raman peak for the films with higher Ge content becomes to be narrower (Fig. 7c). However, for T_A_ = 800 °C, significant contribution of amorphous Ge signal is still observed for the samples with high Ge content.

Thus, the analysis of Raman scattering data allows to conclude that the crystallization of Ge-ncs occurs at T_A_ = 600–700 °C, demonstrating the trend to the lowering of crystallization temperature when the Ge content increases. However, Raman scattering spectra could not provide information about the evolution of ZrO_2_ host. For this purpose, the XRD study was performed for the same set of Ge-free and Ge-rich-ZrO_2_ samples. Besides, information about Ge phase crystallization was also extracted and compared with Raman scattering data.

### X-ray Diffraction Study of Pure and Ge-Rich ZrO_2_ Materials

**Fig. 8:**
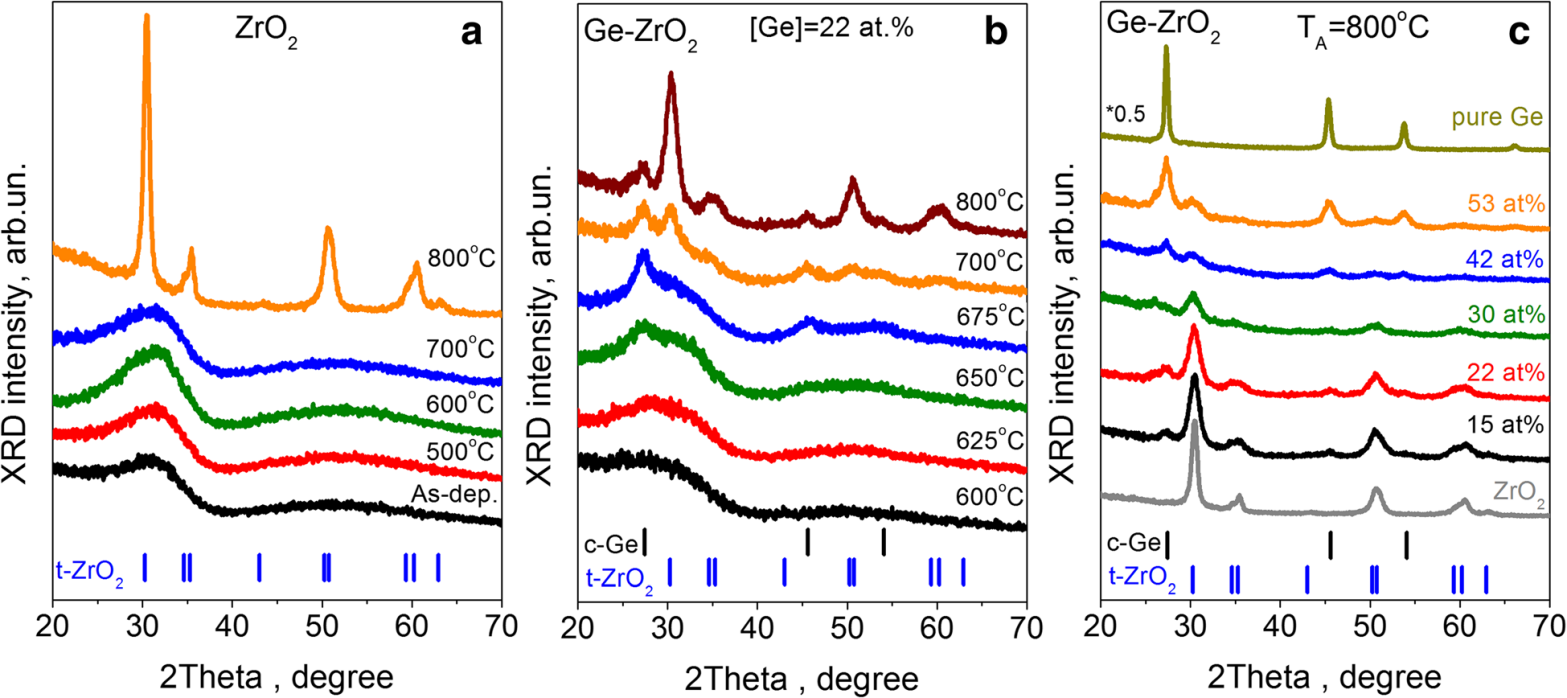
Evolution of XRD patterns for pure ZrO_2_ (**a**) and Ge-rich-ZrO_2_ (**b**, **c**) samples with annealing temperature (**a**, **b**) and Ge content in the films (**c**). Annealing temperature and Ge content are shown in the figures

Figure 8 depicts the evolution of XRD patterns of pure and Ge-rich ZrO_2_ samples. Pure ZrO_2_ films conserve their amorphous nature up to T_A_ = 700 °C, whereas annealing at higher temperature leads to a crystallization of the films (Fig. 8a). An appearance of strong reflections at 2Θ ≈ 30, 35, 50, and 60° testifies the formation of tetragonal ZrO_2_ phase at T_A_ = 800 °C (Fig. 8a). This allows concluding that temperature of crystallization of pure ZrO_2_ films is in the range of 700–800 °C.

Besides the formation of Ge-ncs in Ge-ZrO_2_ samples, these latter showed some lowering of crystallization temperature of ZrO_2_ host. As one can see from Fig. 8b, XRD patterns selected for Ge-rich films with [Ge] = 22 at.% exhibit two broad bands peaked at 2Θ ≈ 28° and 2Θ ≈ 50° for T_A_ ≤ 625 °C. These peaks stem from amorphous Ge and ZrO_2_. After annealing at T_A_ = 650 °C, a peak at 2Θ ≈ 27° appeared. It corresponds to the reflection from (111) Ge family planes and testifies not only the formation of Ge phase via phase separation, but also an appearance of some amount of Ge nanocrystallites. Thermal treatment at T_A_ = 675 °C leads to the increase of the peak magnitude as well as to the development of two additional reflexes at 2Θ ≈ 45° and 2Θ ≈ 55°, that are the signatures of (222) and (333) reflections of nanocrystalline Ge (Fig. 8b). The higher T_A_ results in the enhancement of all Ge-related reflexes giving the evidence of pronounced Ge phase crystallization. This data are in a good agreement with Raman scattering ones (Fig. 7).

It is worth to point that whatever Ge content in the Ge-ZrO_2_ films, the Ge crystallites form at higher T_A_ than the temperature of the crystallization of pure Ge film (Fig. 8c). However, the peak position of broad band in the range of 2Θ ≈ 26–31° shifts gradually from about 30.8° to 26.8° that demonstrates an increase of Ge phase contribution in the film structure.

The evolution of XRD patterns with T_A_ showed also that an annealing at T_A_ = 700 °C stimulated an appearance of XRD peaks at 2Θ ≈ 30.4° and 50.2°, corresponded to the reflections from (111) and (220) family planes, respectively. For T_A_ = 800 °C, these peaks became to be stronger and narrower, being accompanied by the appearance of additional peaks in the range of 2Θ ≈ 59–64° (Fig. 8b). Analysis of these XRD patterns gives the evidence of the formation of tetragonal ZrO_2_ phase.

When Ge content increases, the intensity of ZrO_2_-related peaks decreases, followed by their broadening (Fig. 8c). This means that for the samples with higher Ge content, the crystallization of ZrO_2_ phase sets in at higher T_A_ that demonstrates the possibility to form Ge crystallites in amorphous ZrO_2_ host. It can be assumed that for the films with high Ge content the phase separation was uncompleted and some residual Ge ions are still incorporated in Zr-O-Ge bonds. This fact is supported by the FTIR spectra of the samples annealed at 800 °C (Fig. 6). They showed the vibration band in 460–700 cm^−1^ range that is featureless and broader than that of pure ZrO_2_ films. Thus for the films with higher Ge content, either higher T_A_ or longer annealing time (more than 30 s used in present study) are required for complete phase separation.

### General Remarks About Phase Separation Process

The Ge-rich ZrO_2_ ternary compounds are usually considered to be a mixture of GeO_2_ and ZrO_2_ unit cells as a (GeO_2_)_x_(ZrO_2_)_1-x_. In consequence, their decomposition stimulated by thermal treatment on GeO_2_ and ZrO_2_ phases is expected. However, if Ge-rich compound suffers from the lack of oxygen ions, phase separation can have more complex behavior. To get insight on this process in our samples, one can take into account chemical properties of Ge and Zr ions, Ge-O and Zr-O bonds as well as thermodynamic parameters of related oxides summarized in Table 4.

**Table 4:** Chemical properties of Ge, Zr, and O and thermodynamic parameters of related oxides

Parameter	Element		
	Ge	Zr	O
Ionic radius, Å	0.53 [Ge^4+^]	0.79 [Zr^4+^]	1.40 [O^2−^]
Atomic radius, Å	1.23	1.60	0.66
Electronegativity, χ	2.01	1.33	3.44
Electronegativity difference upon bond formation, χ_M_-χ_O_	1.43	2.11	0
Coordination number in the M-O bond	4	7	-
Type of M-O bond	covalent polar	ionic	-
Length of M-O bond, Å	1.77	2.13	-
Standard molar enthalpy of the oxide formation at 298.15 K, Δ_f_H^0^ kJ/mol	−261.9 (GeO) −580.0 (GeO_2_)	−1100.6 (ZrO_2_)	-
Standard molar Gibbs energy of the oxide formation at 298.15 K, Δ_f_G^0^, kJ/mol	−237.2 (GeO) −521.4 (GeO_2_)	−1042.8 (ZrO_2_)	-

It is known that thermal stability of oxide-based material depends on the coordination number of ions, M-O bond lengths and their nature (ionic or covalent). The materials with higher coordination number, shorter M-O bond length and covalent nature of this bond demonstrate usually thermal stability.

The nature of M-O bonding is determined by the difference in the electronegativity of elements (χ) composed this bond. When χ_M_-χ_O_ = 0–0.2, the bond is covalent nonpolar, while for χ_M_-χ_O_ = 0.3–1.4 it is covalent polar. For χ_M_-χ_O_ ≥ 1.5, the bond has ionic character. Taking into account the properties of Ge and Zr ions (Table 4), one can see that the Zr-O bond is ionic one, whereas Ge-O bond is covalent polar. It is worth to note that the ionic strength increases with the increase of the X_M_-X_O_ difference, for covalent bonding this relation is opposite.

Taking into account the molar enthalpy and Gibbs energy for ZrO_2_, GeO_2_ and GeO (Table 4), one can assume that upon thermal treatment of Ge-rich ZrO_2_ materials the formation of ZrO_2_ phase is most favorable. This means that this phase will form at first upon thermal treatment. However, its crystalline type can be dependent on the appearance of pure Ge and/or Ge oxide phases.

As it was mentioned above, pure ZrO_2_ films can crystallize upon growing process and/or under thermal treatment. It was shown that doping with [Ge] = 12.5 at.% allows to shift crystallization temperature of ZrO_2_ to higher values as well as to stabilize ZrO_2_ tetragonal phase [12, 27]. Our data show that pure ZrO_2_ films can conserve their amorphous nature up to 700 °C (Fig. 8a). The crystallization of ZrO_2_ occurs at T_A_ = 800 °C and results in the formation of tetragonal ZrO_2_ domains with mean size of about 10 nm. It was reported that small ZrO_2_ grains crystallized usually in tetragonal and/or cubic phase [39]. Thus, the observation of tetragonal ZrO_2_ grains in our films can be expected.

The Ge-rich ZrO_2_ films showed the formation of tetragonal ZrO_2_ phase at lower temperature (~700 °C) (Fig. 8b). The mean size of ZrO_2_ domains was found to be about 6 nm that can be one of the reasons of tetragonal phase formation. Another argument for this phase formation is the difference in the Ge-O and Zr-O bond lengths. In the ordered structure, Ge ions adopt a 4-fold coordination leaving eightfold coordination to the larger cations, and the pattern for cation partition is layer-like. When Ge cations incorporate into ZrO_2_ host, the formation of Ge-O bonds will cause the stretching out of Zr-O ones [17] because the Ge-O bonds are shorter and stronger than Zr-O distances. Upon annealing this bonding anisotropy will result in the higher tetragonality.

One more argument for the lowering of ZrO_2_ crystallization temperature is metallic behavior of Zr ions themselves. Their presence makes weaker Ge-O bonds and, thus, stimulates their breaking, followed by the formation of pure Ge phase. Finally, the depletion of ZrO_2_ by Ge will give impact to the ZrO_2_ crystallization. At the same time, the formation of Ge crystallites will bring additional stretching of ZrO_2_ phase due to larger lattice parameter of Ge crystallites in comparison with that of ZrO_2_. Thus, such stretching of ZrO_2_ phase will favor the stabilization of its tetragonal modification.

As it was mentioned above, from thermodynamic point of view the formation of ZrO_2_ phase is preferable (Table 4). This means that Ge-related phase will appear either as GeO_2_ (for the case of (GeO_2_)_x_(ZrO_2_)_1-x_ materials) or as GeO_y_ (for the case of lack oxygen). In the latter case, the formation of Ge nanoclusters will occur via reaction 2GeO_y_ → (2-y)Ge + yGeO_2_. However, GeO_2_ is known to be transformed at T_A_ = 420 °C via reaction GeO_2_ + Ge → 2GeO followed by desorption of volatile GeO at higher T_A_ (~450-500 °C) [22, 40]. Thus, the formation of Ge-ncs will depend not only on the Ge content in the films but also on the competition between Ge-ncs and GeO formation during annealing. Since the Gibbs energy is lower for GeO_2_ than that for GeO, one can expect that this competition will be shifted towards Ge-ncs formation when Ge content is higher.

It is worth to note, that the presence of Ge crystallites of large amount in our samples annealed at T_A_ = 700 °C allowed to suppose that the annealing regime caused GeO formation in Ge-rich ZrO_2_ films differs significantly from that described for pure GeO_2_ layers [22, 40]. However, for T_A_ = 800 °C, the redistribution of Ge ions over film volume as well as the enrichment of capping SiO_2_ layers with Ge observed by AES method can be explained by the significant contribution of GeO formation upon annealing.

Indeed, all the samples were capped with SiO_2_ layers that can prevent significant outward diffusion of Ge from the layers via sublimation of GeO [22]. However, AES data showed the decrease of Ge content over film volume. Thus, the increase of T_A_ up to 800 °C results in the strong competition between two processes (i.e., the Ge-ncs and GeO formation) that is important for the films with lower Ge content. In this regard, to achieve higher amount of Ge-ncs for the films with [Ge] ≤ 30 at.%, the optimization of annealing treatment can be performed via optimization of annealing time. This work is in progress.

## Conclusions

This work shows the utility of RF magnetron sputtering for the fabrication of undoped and Ge-doped ZrO_2_ films with required properties. The Ge content in the films was controlled via the RFP_Ge_ value at other constant deposition parameters. Rapid thermal treatment was used to form Ge crystallites in the films.

The as-deposited pure ZrO_2_ and Ge-ZrO_2_ films and those annealed at T_A_ ≤ 600 °C demonstrate amorphous nature. Annealing at higher T_A_ of Ge-rich ZrO_2_ films stimulates a phase separation and the formation of Ge-ncs. The mechanism of phase separation was discussed.

The crystallization of Ge-ncs sets in at T_A_ = 640–700 °C and depends on the Ge content: the higher the Ge content, the lower is the Ge crystallization temperature. The ZrO_2_ matrix crystallizes at higher temperature (680–700 °C) than the Ge phase, but its crystallization temperature is lower than that of pure ZrO_2_. An appearance of tetragonal ZrO_2_ phase is observed. The technological window to form Ge crystallites in amorphous ZrO_2_ host is demonstrated.
